# A novel CAT method for QoL screening: proof-of-principle study with comparisons to standard methods

**DOI:** 10.1007/s11136-025-04035-5

**Published:** 2025-07-26

**Authors:** Anastasios Psychogyiopoulos, Niels Smits, L. Andries van der Ark

**Affiliations:** Research Institute of Child Development and Education, University of Amsterdam, Postbus 15780, 1001 NG Amsterdam, The Netherlands

**Keywords:** Adaptive screening, Computerized adaptive test, Depression, LSCAT, Stochastic curtailment, Decision tree

## Abstract

**Purpose:**

This proof-of-principle study investigated a novel Computer Adaptive Testing (CAT) method termed Latent-class and Sum score based Computerized Adaptive Testing (LSCAT), developed for screening purposes. LSCAT was assessed for its ability to accurately predict depression symptoms during health-related quality of life (HR-QoL) screenings.

**Methods:**

LSCAT’s performance was compared with two benchmark CAT methods, Stochastic Curtailment (SC) and Decision Tree based Computer Adaptive Testing (DTCAT), using data from the Patient Health Questionnaire-9 (PHQ-9).

**Results:**

LSCAT consistently outperformed both SC and DTCAT in terms of predictive accuracy, achieving the lowest rates of Type I error. Furthermore, LSCAT’s Type II error rates were at least as low as those of SC and significantly lower than those of DTCAT across all simulation scenarios.

**Conclusion:**

These results suggest that LSCAT is a promising method for developing valid and efficient screening tools in HR-QoL research and practice.

## Introduction

Patient-reported instruments are mainly used to measure health-related quality of life (HR-QoL). Less frequently, but not less importantly, such instruments are used for screening, wherein patients’ responses to a short questionnaire are used to predict whether they require a more comprehensive clinical assessment or an intervention [12, 21]. Screening can be valuable because providing comprehensive assessments or interventions only to patients who need them is both cost-effective and time-efficient. For example, to determine whether a more comprehensive assessment is necessary for the clinical diagnosis of depression, respondents may complete the short PHQ-9 questionnaire, in which patients with a sum score of at least 20 are labeled ’severely depressed’. Having a sum score of at least 20 may serve as a cutoff for clinical therapy [15]. Efficient screening improves communication between respondents and healthcare providers, facilitates decision-making, and eventually enhances patient quality of life [9, 36]. The key to an efficient screening is finding the right balance between predictive validity and administration time.

In clinical research and practice, long questionnaires can be a burden for patients and healthcare providers [24], leading to the infrequent use of such assessments [23]. Despite the development of shorter versions of existing instruments to obtain as much information as possible in the shortest possible time (see, [17], for a review), the administration of short questionnaires with five to 14 items may be difficult in practice [25]. This highlights the necessity for even briefer tools, the so–called ultra–short tests (1–4 items, $<2$ minutes). Examples include the PHQ-2 [20], which uses the first two of nine items from the PHQ-9 [15] for depression screening, and the GAD-2 [16], which uses two of seven items from the GAD-7 [34] for general anxiety disorder screening.

Whereas the use of ultra-short screening tools may be useful in situations with very short time, test shortening may come at the expense of predictive validity. Balancing test length and predictive validity is critical for a useful screener. However, excessive shortening may reduce predictive validity to unacceptable levels [14, 17], potentially leading to suboptimal outcomes.

The development of a screener involves a large-scale study to identify a relatively small set of items whose sum score can accurately predict the outcome of a comprehensive assessment or intervention. Subsequently, a cutoff value is established for the sum score to optimally predict a respondent’s *status*; that is, whether or not the respondent is eligible for the full assessment or intervention. Because only the cutoff score is used to determine a respondent’s status, a screener must be highly reliable around the cutoff score, whereas reliability for other sum score values is less critical. Note that the majority of tests are developed for *measurement* of the general population and require high reliability for all test scores (for a discussion see [31]).

The response burden can be reduced further by only administering the items in the screener that are necessary to predict a respondent’s status, which is the topic of this paper, and several methods are available to accomplish this.

Computer adaptive testing (CAT) is a popular method to reduce test length while gaining as much information as possible. Each respondent answers a different set of items from a large pool, allowing for efficient measurement in less time. Traditional CAT applications are based on item response theory (IRT) [41, 43], here referred to as IRTCAT, which is used extensively in many different areas within the field of HR-QoL [2, 6, 37]. However, in practice questionnaire data do not always conform to the statistical assumptions of IRT [3, 28, 39, 44]. Moreover, while IRT can be an excellent tool for measurement purposes, it is suboptimal for prediction [11, 31]. The main reason is that an IRT model fits the data well when the items are homogeneous—a condition necessary for accurate measurement—but item homogeneity generally reduces predictive validity [4, 31]. As a result, accurate prediction may require an excessive number of items, even when such accuracy is unnecessary for distinguishing individual test results [11]. These limitations render IRTCAT unsuitable for screening purposes; therefore, other methods are needed to ensure accurate and precise predictions in minimal time.

Stochastic curtailment (SC, [4]) aims to provide an efficient assessment for status prediction. Under SC, the test items are administered until there is sufficient confidence about the respondent’s status. Typically, sufficient confidence is achieved when the sum of the administered items exceeds a pre-specified cutoff [4, 5]. SC has been shown to reduce the average number of administered items by over $40\%$ [32]. However, SC is an *early stopping algorithm*: It does not support dynamic item selection but administers items in a fixed order until a stopping rule is met or all items have been administered. Therefore, some informative items may be excluded or appear too late in the assessment, which may cause the screening process to be unnecessarily long or could lead to less favourable predictions.

The basis of Decision Tree based CAT (DTCAT) is a tree structure resulting from Classification and Regression Tree methods [1] that select optimal predictor variables and associated cutoff scores in each node to create subgroups that differ maximally in the outcome variable. Paths from the root to the end nodes allow for efficient testing by administering only the items encountered along a path. For DTCAT construction, a calibration dataset is used in which the item scores are treated as predictor variables. The dependent variable is either internal (i.e., an item–based score), such as a scale score [45], or external, such as a diagnosis by a therapist [8, 10, 11]. In the application phase, the tree structure is used as an adaptive test: Depending on the answers given, each respondent follows a specific path, ultimately leading to a prediction of the outcome. DTCAT seeks high prediction accuracy with minimal items per respondent and has proven effective [8, 10, 11, 45]. However, because DTCAT only includes a small number of all possible paths (i.e., response patterns), it is not truly adaptive, potentially omitting predictive items and leading to suboptimal screening.

The objective of this paper is to present the potential usefulness of a new CAT method, called Latent-class and sum score based Computerized Adaptive Testing (LSCAT). Previous research has shown the effectiveness of LSCAT in measurement contexts: LSCAT demonstrated measurement precision comparable to that of IRTCAT on real data following a multi-modal distribution [26]. It was also suggested to be more efficient for respondents with scores in the lower and upper extremes [26]. The last finding may be of particular importance for HR-QoL research, as it demonstrates the potential of LSCAT for accurate and precise predictions in screening tests. This study aims to showcase this potential by putting LSCAT into practice for the first time on a predictive task: screening in the context of HR-QoL.

This study hypothesized that LSCAT would be particularly useful for screening because it addresses certain limitations inherent to the adaptive methods described above. First, LSCAT has the potential to be used for prediction, and second, it employs dynamic item selection [26]. The current paper aims to demonstrate the usefulness of LSCAT in a proof-of-principle study, using SC and DTCAT as benchmarks. To enhance readability, a conceptual description of LSCAT is provided in the next section, and the statistical model is deferred to Appendix A.

## A conceptual description of LSCAT

Van der Ark and Smits [40] introduced a typology of CAT by engine (i.e., model underlying the CAT) and score (i.e., the score used to report individual outcomes). For example, traditional CAT uses an IRT model for an engine and the latent trait estimate for a score. LSCAT fits in this typology, using the unrestricted latent class model (ULCM, [13]) for an engine and the sum score for a score [30]. With the ULCM being one of the most flexible measurement models, and the simple sum score adhering to the practical use of screeners, LSCAT can be used in both measurement and prediction settings employing a dynamic item administration for an adaptive test that is fully tailored to the individual. In the current application of LSCAT we use the ULCM as an engine and the respondent’s status (‘eligible’ or ‘not eligible’) as a score. The probability that the sum score is at least as large as the cutoff value (i.e., status is ‘eligible’) is denoted *eligibility probability*.

As is common in all CAT, LSCAT has a calibration and an administration phase. For calibration, a large sample of respondents with item scores on the full test is used to estimate an ULCM. From the ULCM, the probabilities of all possible response patterns can be derived. By summing up the appropriate response-pattern probabilities, the eligibility probability can be obtained (Appendix A). In the administration phase, the items are presented to the respondent one at a time. After an item has been answered, the estimated response-pattern probabilities are updated, by simply setting the probability to zero for all response patterns that have become obsolete in presence of the newly obtained item score, and normalizing the probabilities of the response patterns that are still feasible. The updated estimates of the response-pattern probabilities are then used to update the estimate of the eligibility probability. When the probability that a respondent has a certain status (either ‘eligible or ‘not eligible’) exceeds a pre-specified threshold, referred to as a *stopping criterion* (also, see Appendix A), the test halts, assigning this status as the final decision for the respondent. For details on item selection, we refer to Van der Ark and Smits [40].

## Methods

The present study employs a post-hoc simulation methodology, which involves conducting CAT simulations on existing complete test data (see e.g., Finkelman et al. [4]) and treating the respondent’s observed item scores and sum score as the true values. Consequently, in the simulations, the respondent’s true status, ‘eligible’ or ‘not eligible’, is known. The predictive validity of an adaptive algorithm may be illustrated by comparing the true status with the predicted status. LSCAT was evaluated by studying the degree of concordance between the predicted and true status in a number of different conditions. In the post-hoc simulation, the performance of LSCAT was compared to that of two benchmark methods: SC and DTCAT.

The post-hoc simulation consisted of four steps: First, the data were randomly split into a calibration and a validation sample. Then, each method’s algorithm was trained using the calibration sample. Subsequently, the trained methods were applied to the validation sample, producing a predicted status for each respondent in the validation sample. Finally, for each method, the predicted validity was established by comparing the respondents’ predicted and true statuses.

The three methods were also compared to a lower benchmark method, called *base rate* in which respondents were assigned randomly to the eligible status with a probability equal to the base rate in the calibration data.

### Data

We used item responses to the nine-item Patient Health Questionnaire (PHQ-9, [15]) which is a popular self-report questionnaire [18] assessing the presence or absence of 9 depression symptoms in the past two weeks using items measured on a four-point Likert scale: ’not at all’ (0), ‘several days’ (1), ‘more than half the days’ (2), and ‘nearly every day’ (3). With a sum score ranging from 0 to 27, cutoff values of 5, 10, 15, and 20 are used as thresholds for *mild*, *moderate*, *moderately severe* and *severe* depression symptoms, respectively, while values below 5 indicate absence of depressive symptoms [15]. This study looked at two levels of depression: mild and moderate.

A total sample of 20, 685 individuals, representative of the US general population, was used from the National Health and Nutrition Examination Survey (NHANES). The data were randomly split into a calibration dataset ($N_c = 10,343$, $M = 3.152$, $SD = 4.231$) and a validation dataset ($N_v = 10,342$, $M = 3.149$, $SD = 4.247$). The sum scores showed a skewed distribution in both the calibration and validation data sets, resulting in uneven categorizations. In the calibration subset, 24% of respondents were classified as at least mildly depressed (sum score $\ge 5$), and 9% as at least moderately depressed (sum score $\ge 10$). These percentages served as the base rates for random respondent assignment into the two categories. For a more detailed description of the data, we refer to Smits and Finkelman [32].

### Calibration

For LSCAT calibration, a ULCM was estimated using the Bayesian information criterion [29] for selecting the number of latent classes [26]. The eligibility probabilities were derived from the estimated ULCM parameters (Appendix A). For the calibration of SC, a series of logistic regression models was estimated to determine the eligibility probabilities. This resulted in a look-up table indicating when a respondent’s cumulative score was sufficient to either stop the test or proceed to the next item. More details of the implementation can be found in Finkelman et al. [5].

For DTCAT, the process involved training a classification tree (see e.g., Strobl et al. [35]). Unlike the other two methods, the calibration process for DTCAT requires pre-specifying all paths a hypothetical respondent may follow before a decision on the predicted status is made. This was performed by specifying a single decision tree on the calibration sample, as proposed by [8, 11].

### Post-hoc simulations

#### Independent variables

Simulations were conducted in a 2 (Cutoff) $\times$ 2 (Stopping criterion) $\times$ 3 (Method) cross-factorial design. Factor Cutoff had levels ’at least 5’ (i.e., at least mildly depressed) and ’at least 10’ (at least moderately depressed). Factor Stopping criterion was defined as the average number of items administered when the LSCAT stopping criterion was set at $c =.95$ and $c =.99$, respectively (see Appendix A). Because the benchmark methods SC and DTCAT do not define stopping criteria in terms of *c*, these methods were configured to, on average, use the same number of items in a design cell as LSCAT. Lastly, factor Method had three levels: LSCAT, SC, DTCAT.

*Efficiency*, the average number of items administered to a single respondent, was fixed across the three methods. First, the average number of items administered for LSCAT under a certain stopping rule was computed. Then, by a priori fine-tuning the stopping rules of SC and DTCAT, a stopping rule was selected for these methods that yielded the same average of administered items (see, Appendix B). Fixing efficiency, a common practice in the CAT literature, allows a fair comparison of the methods’ predictive validity under an equal response burden [33, 40].

#### Dependent variables

The discrepancy between respondents’ true and predicted statuses was studied with four indicators of predictive validity. First, the Type I error rate was defined as the proportion of false positives over actual negatives (Type I error rate $= \frac{FP}{TN + FP}$). Second, the Type II error rate was defined as the proportion of false negatives over actual positives (Type II error rate $= \frac{FN}{TP + FN}$). Third, accuracy was defined as the proportion of true positives (i.e., ‘hit‘) plus true negatives (i.e., correct rejection) over actual positives and negatives ($Accuracy = \frac{TP + FN}{TN + FP + TP + FN}$). In situations where the dataset is considered imbalanced (i.e., uneven category distribution), the *Accuracy* measure may be biased towards the majority status. Therefore, Cohen’s $\upkappa$ can be used as a complementary measure [46] and can be interpreted using the following rules of thumb: $0-0.20$ as ‘none to slight‘, $0.21-0.40$ as ‘fair‘, $0.41-0.60$ as ‘moderate‘, $0.61-0.80$ as ‘substantial‘, and $0.81-1$ as ‘almost perfect‘ [22]. Finally, the classification errors were studied as a function of the full administration sum scores. It was chosen to report on this outcome only when methods performed similarly on the dependent variables.

### Software

The analysis was performed in R [27] using R studio. For LSCAT, the ULCM estimation was performed using the poLCA package [19], and the simulation was conducted using the authors’ own code. Both calibration and simulation for DTCAT were conducted using the rpart package [38], whereas for SC custom code in R was used. All computer code is available on OSF: https://osf.io/g5hz7/.

## Results

**Table 1:** Efficiency fixed to yield an equal number of administered items on average across methods for a given combination of cutoff and stopping criterion

Cutoff	Stopping criterion	Method	Administered Items		
			*M*	*SD*	Range
$\ge 5$	.95	LSCAT	3.248	1.931	1–9
		SC	3.863	1.646	1–8
		DTCAT	3.982	0.539	3–5
	.99	LSCAT	4.613	1.968	2–9
		SC	4.948	1.761	1–9
		DTCAT	4.780	0.850	3–6
$\ge 10$	.95	LSCAT	1.789	1.714	1–9
		SC	1.786	1.775	1–9
		DTCAT	2.053	0.224	2–3
	.99	LSCAT	3.045	1.919	2–9
		SC	3.002	1.836	2–9
		DTCAT	3.000	0.296	2–4

*Note. LSCAT =* Latent-class sum score computerized adaptive testing; *SC* = Stochastic curtailment; *DTCAT =* Decision tree based computer adaptive testing

Table 1 (fourth column) shows results for fixing the efficiency (i.e., average number of administered items) across all methods. Idiosyncracies of the data precluded exactly equal numbers. Whereas the averages differed only in the second decimal in the condition with a cutoff of 10 and $c=.99$, in the three other combinations the methods differed a bit more; most notably in the condition of a cutoff of 5 and $c=.95$, in which SC, compared to LSCAT, used 0.615 additional items on average, putting LSCAT in a slightly unfavorable position for cross-method comparison. However, as this study aims to illustrate the usefulness of LSCAT the approximately equal average number of administered items was deemed a sound basis for comparing the methods. In addition, whereas LSCAT and SC used the full range of available items, DTCAT exhibited the lowest *SD*, as reflected in its narrow range of administered items.

**Table 2:** Post-hoc simulation results

Cutoff	Stopping criterion	Method	Type I ER	Type II ER	Accuracy	$\upkappa$
$\ge 5$	-	*Base rate*	0.239	0.769	0.633	$-$0.008
	.95	LSCAT	**0**.**003**	0.069	**0**.**981**	0.947
		SC	0.008	**0**.**043**	0.984	**0**.**955**
		DTCAT	0.030	0.164	0.938	0.826
	.99	LSCAT	**0**.**000**	0.013	**0**.**997**	**0**.**991**
		SC	0.004	**0**.**012**	0.994	0.984
		DTCAT	0.042	0.093	0.946	0.854
$\ge 10$	-	*Βase rate*	0.093	0.920	0.837	0.023
	.95	LSCAT	**0**.**001**	**0**.**128**	**0**.**989**	**0**.**921**
		SC	**0**.**001**	0.165	0.985	0.896
		DTCAT	0.017	0.315	0.958	0.709
	.99	LSCAT	**0**.**000**	**0**.**024**	**0**.**998**	**0**.**985**
		SC	0.001	0.050	0.995	0.967
		DTCAT	0.023	0.225	0.960	0.745

*Note.* For each combination of Cutoff and Stopping criterion, the results of the best-performing method are printed in boldface. ER = error rate; $\upkappa$ = Cohen’s kappa; LSCAT = Latent-class sum score computerized adaptive testing; SC = Stochastic curtailment; DTCAT = Decision tree based computer adaptive testing

Table 2 shows that LSCAT consistently outperformed the benchmark methods, SC and DTCAT, on all indicators of predictive validity. Specifically, LSCAT had the lowest Type I error rate and the highest *Accuracy* in all conditions. It also demonstrated the best results for the Type II error rate and Cohen’s $\upkappa$ in three out of four conditions. Although SC exhibited somewhat better results for the Type II error rate and $\upkappa$ in one scenario (cutoff value of ‘at least 5‘ and stopping criterion of .95), this was in the situation where equating the number of items was less successful and SC used 0.615 items more than LSCAT, on average (see, Table 1). Notably, LSCAT achieved an almost perfect Cohen’s $\upkappa$ in all design cells. In contrast, DTCAT consistently showed the least favourable results in all conditions and produced considerably higher Type II error rates, particularly under the higher cutoff. Examining the benefits of CAT methods compared to random assignment using the *base rate* method confirms that CAT methods offer advantages, as random assignment generally resulted in low indicators of predictive validity.

**Fig. 1:**
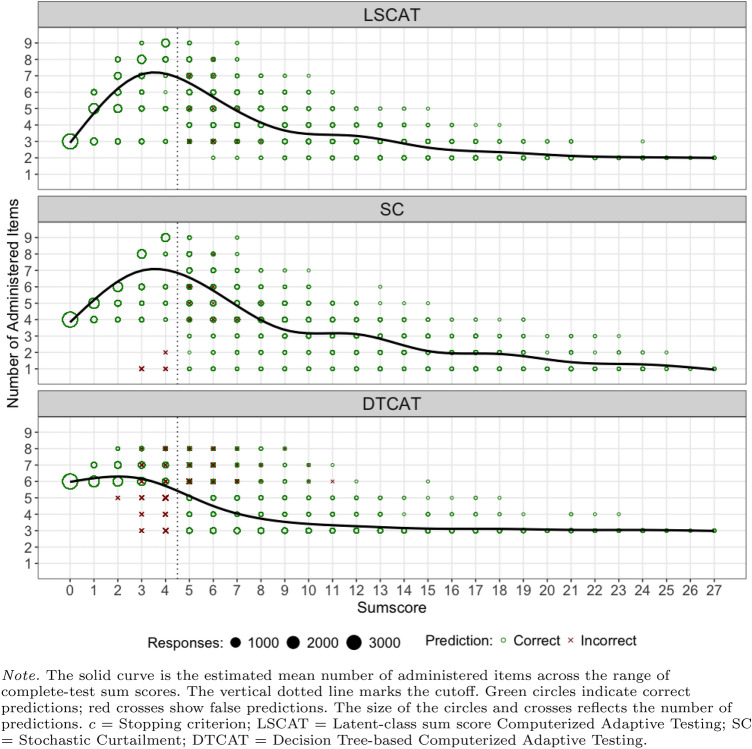
The distribution of number of administered items as a function of the complete–test sum score for cutoff $\ge 5$ and $c =.99$

In a post-hoc analysis, the three methods were further investigated. For the condition with cutoff $\ge 5$ and $c=.99$, Fig. 1 shows for each of the three methods the distribution of the administered number of items (vertical axis) for each complete-data sum score (horizontal axis). These distributions were similar for LSCAT and SC: As expected, both the number of misclassifications and the highest number of administered items were close to the cutoff. For DCAT, the misclassifications were more dispersed, and the highest number of administered items was in the lower ranges of complete-test sum scores. Figures for other cutoff values and values of *c* are available on OSF.

## Discussion

In this study, LSCAT was introduced as a method for constructing efficient screeners. A post-hoc simulation study was performed to illustrate its usefulness when screening for the presence of depression symptoms in the general population. LSCAT’s predictive validity was compared with that of two other adaptive methods previously shown to be efficient and effective, SC [4, 5, 32] and DTCAT [7, 8]. LSCAT demonstrated high accuracy with minimal error rates across various simulation conditions. With respect to predictive validity, LSCAT exceeded or matched SC, and outperformed DTCAT. Therefore, the proof of principle was successful, but without further research, the results from this paper cannot be generalized to other screeners.

The next step would be a comprehensive study to generalize the results from this paper and address the remaining questions. First, it would be valuable to investigate further whether SC, a non-adaptive method, will also be successful for screeners other than the PHQ-9. A possible explanation for the favorable results for the PHQ-9 could be its item order. In this study, the most informative items for LSCAT were items 2, 3 and 4. As a result, the first four items of LSCAT and SC are very similar for many respondents, and it is not surprising that the predictive validity of LSCAT and SC is also similar.

Second, it would be interesting to investigate why DTCAT was relatively unsuccessful in this paper. The popular DTCAT method used in this study [8] uses a single tree, which is pruned to consist of two equally sized branches. Adjusting the pruning process could potentially enhance its performance. In addition, replacing a single tree with a random forest may improve the results, yet random forests are still under development for CAT [7].

Third, LSCAT is a work in progress. This paper provides a proof of principle that LSCAT can be used for prediction and can successfully reduce the number of items in a screening procedure. However, further research is needed to examine its performance under various settings and conditions. In addition, while LSCAT works smoothly for screeners with a limited number of items, computer memory issues arise when the item bank exceeds approximately 15 items–an issue we are currently investigating. Finally, although our R code is freely available on the Open Science Framework, implementation in user-friendly software is necessary before LSCAT can be used at a larger scale. One of the desiderata for such software would be the ability to recalibrate the latent class model online when new responses are collected or when items are added or removed.

This proof-of-principle study marks an initial step, demonstrating the potential of a flexible and efficient screening method for broader use in QoL research and practice. Practical implementation of the proposed procedure requires three additional steps: LSCAT for prediction should be scalable to large item banks. While this is not necessary for brief screeners such as the PHQ-9, it would broaden the range of applications. LSCAT for prediction should be made available in user–friendly software. We are currently developing R code for an upcoming package that will be openly available. Researchers intending to apply LSCAT must provide a sufficiently large training dataset. As with other forms of computerized adaptive testing, LSCAT requires a dataset for calibration of the latent class model. The first two steps are within our scope; the third depends on the researchers who implement the method.

## Data Availability

The dataset used in the current article is available at https://osf.io/g5hz7/.

## Appendix A LSCAT technical details

Suppose a test has $J$ items, each with $m+1$ ordered scores $0, 1, \dots , m_j$. Let $X_j (j = 1, \dots , J)$ be a random variable denoting the score on item $j$ with realization $x_j \in \{0,1,\dots ,m\}$, and let $\Theta$ be a discrete latent variable with $K$ classes ($\uptheta _1, \dots , \uptheta _K$). The unrestricted latent class model (ULCM) assumes independence of the *J* item scores given latent class membership; that is,

A1 $$\begin{aligned} P(x_1,\dots , x_J \mid \uptheta _k) = \prod _{j=1}^J P(x_j \mid \uptheta _k). \end{aligned}$$

Using Bayes’ rule, it follows that

A2 $$\begin{aligned} P(x_1,\dots , x_J, \uptheta _k) = P(\uptheta _k)\prod _{j=1}^J P(x_j \mid \uptheta _k), \end{aligned}$$

and

A3 $$\begin{aligned} P(x_1,\dots , x_J) = \sum _{k=1}^K P(\uptheta _k)\prod _{j=1}^J P(x_j \mid \uptheta _k). \end{aligned}$$

Equation A3 shows that response pattern probability $P\left( x_{1},\dots ,\ x_{J} \right)$ can be written as a function of the *class weights* $P\left( \uptheta _{k} \right) \ (k = 1,\dots ,\ K)$ and *response function* $P(x_{j}|\uptheta _{k})$ $(j = 1,\ \dots ,\ J;\ k = 1,\dots , K)$, which are the parameters of the ULCM.

In the calibration stage, the class weights and response functions are estimated using a software package for latent class analysis, such as poLCA [19] or Latent GOLD [42], and the response-pattern probabilities are estimated by plugging the estimated class weights and response functions into Eq. A3. Subsequently, the estimated response-pattern probabilities are collected in a vector $\widehat{\varvec{\uppi }}=\left[P(0,\ 0,\ \dots 0),\ \dots ,\ P(m,\ m,\ \dots ,\ m) \right]^{\mathbf {\prime }}$. Next, the response patterns are divided into two mutually exclusive and exhaustive sets: A set of response patterns yielding sum scores below the cutoff ($S_{0}$, status ‘not eligible’), and a set of response patterns yielding sum scores equal to or above the cutoff ($S_{1}$, status ‘eligible’). The probabilities for each set of response patterns (i.e., $P(S_{0}$) and $P(S_{1}$)) are estimated by summing the appropriate response-pattern probabilities. Let ${\textbf{d}}_{1}$ be indicator vector where a 1 indicates that the corresponding estimated response-pattern probability in $\widehat{\varvec{\uppi }}$ belongs to $S_{1}$ and 0, otherwise, and let ${\textbf{d}}_{0}$ be indicator vector where a 1 indicates that the corresponding estimated response-pattern probability in $\widehat{\varvec{\uppi }}$ belongs to $S_{0}$ and 0, otherwise, then $P(S_{0})$ and $P(S_{1})$ can be obtained by

A4 $$\begin{aligned} \begin{bmatrix} P(S_{0}) \\ P(S_{1}) \end{bmatrix} = \begin{bmatrix} {\textbf{d}}_{0}^{\prime } \\ {\textbf{d}}_{1}^{\prime } \end{bmatrix}\widehat{\varvec{\uppi }}. \end{aligned}$$

In the administration stage, for a certain respondent $P(S_{0}$) and $P(S_{1}$) in Eq. A4 are used as initial estimates. In a cyclic procedure, first the most informative item is selected (see Van der Ark & Smits, [40], for details), and presented to the respondent. Once an item has been answered, the response patterns that do not align with the respondent’s given response become inadmissible, and the indicators of these response patterns in ${\textbf{d}}_{0}$ and ${\textbf{d}}_{1}$ are set to zero. Applying Eq. A4 using the updated vectors ${\textbf{d}}_{0}$ and ${\textbf{d}}_{1}$ and normalizing the results yields an update of $P(S_{0})$ and $P(S_{1})$. In a cyclic procedure, the respondent is presented the post informative items from the remaining set of items, and each time $P(S_{0})$ and $P(S_{1})$ are updated. Following Van der Ark and Smits, the procedure stops if $\max \left( P\left( S_{0} \right) ,\ P(S_{1}) \right)> c$, where $c$ is a sufficiently large stopping criterion (e.g., .90, .95 or .99).

## Appendix B

See Table

**Table 3:** Stopping Criteria Used for Early Stopping in SC and for Pruning in DTCAT

Cutoff	Stopping criterion	Method	
		SC	DTCAT
$\ge 5$	.95	$\upgamma = 0.9375$	cp $= 0.0070$ & max depth $= 5$
	.99	$\upgamma = 0.9851$	cp $= 0.0015$ & max depth $= 6$
$\ge 10$	.95	$\upgamma = 0.9600$	max depth $= 3$
	.99	$\upgamma = 0.9850$	max depth $= 4$

Note. LSCAT = Latent-class sum score computerized adaptive testing; SC = Stochastic curtailment; DTCAT = Decision tree based computer adaptive testing; $\upgamma$ = Probability threshold parameter for early stopping in SC; cp = complexity parameter; max depth = maximum number of levels (splits) in a decision tree

3.

## Code availability

The code is available at https://osf.io/g5hz7/.
